# Copy Number Variations in Short Tandem Repeats Modulate Growth Traits in Penaeid Shrimp Through Neighboring Gene Regulation

**DOI:** 10.3390/ani15020262

**Published:** 2025-01-18

**Authors:** Hao Zhou, Guangfeng Qiang, Yan Xia, Jian Tan, Qiang Fu, Kun Luo, Xianhong Meng, Baolong Chen, Meijia Chen, Juan Sui, Ping Dai, Xupeng Li, Mianyu Liu, Qun Xing, Jie Kong, Sheng Luan

**Affiliations:** 1 State Key Laboratory of Mariculture Biobreeding and Sustainable Goods, Yellow Sea Fisheries Research Institute, Chinese Academy of Fishery Sciences, Qingdao 266071, China; zhouhao@ysfri.ac.cn (H.Z.); qianggf1991@163.com (G.Q.); xiayan1207@126.com (Y.X.); tanjian@ysfri.ac.cn (J.T.); fuqiang@ysfri.ac.cn (Q.F.); luokun@ysfri.ac.cn (K.L.); mengxianhong@ysfri.ac.cn (X.M.); chenbl@ysfri.ac.cn (B.C.); chenmjwork@163.com (M.C.); suijuan@ysfri.ac.cn (J.S.); daiping@ysfri.ac.cn (P.D.); lixupeng@ysfri.ac.cn (X.L.); 2022213007@stu.njau.edu.cn (M.L.); 2 Laboratory for Marine Fisheries Science and Food Production Processes, Qingdao Marine Science and Technology Center, Qingdao 266237, China; 3 BLUP Aquabreed Co., Ltd., Weifang 261311, China; xingqun527@163.com

**Keywords:** short tandem repeats, STR, growth trait, penaeid shrimp, molecular breeding

## Abstract

Penaeid shrimp, with its genomes enriched in short tandem repeats (STRs), presents an ideal model for studying the distribution and biological functions of STRs. In this study, we systematically identified and compared STRs across multiple species, confirming a significantly higher prevalence of STRs (26–32%) in penaeid shrimp, which is markedly higher than that observed in other species, such as mammals (1.3–2.1%) and plants (0.21–0.73%). Further analysis utilizing a cohort of 326 Pacific white shrimp revealed a total of 672,507 high-quality STRs evenly distributed across the genome, with a notably lower frequency of SNPs within these STR regions. Focusing on growth traits as a case study, we conducted a genome-wide association study (GWAS) and correlation analyses to identify the regulatory relationship of STRs on complex traits. We discovered 84 STRs that showed a significant association with body weight. Interestingly, eleven of these STRs, with 81% being composed of an A/T base, showed a significant linear correlation with body weight, revealing the key role of A/T-related STRs in shrimp weight regulation. For instance, a significant association and a negative correlation were found between the copy number of the STR [(A)n] at NW_020872788.1:580574 and body weight. The cytokinesis protein 7-like (LOC113800912) gene, which contains this STR in its splice region, exhibits differential expression associated with body weight variation. These findings introduce a model for STR copy number regulation in non-human species, illuminating the influence of STRs on growth traits. It offers a valuable framework for investigating complex traits and the biological functions of STRs in agricultural animals.

## Simple Summary

Penaeid shrimp, with its genomes enriched in short tandem repeats (STRs), presents an ideal model for studying the distribution and biological functions of STRs. In this study, we systematically identify and compare STRs across various species, finding that penaeid shrimp exhibit a markedly higher prevalence of STRs compared to other groups, such as mammals and plants. Subsequent analysis of a cohort of 326 Pacific white shrimp identified 672,507 high-quality STRs, which were evenly distributed across the genome, with a notably lower frequency of SNPs within these regions. Our analyses show that specific STRs, particularly those rich in A/T bases, are significantly associated with body weight. One notable finding is the association of an STR in the splice region of the cytokinesis protein 7-like gene with variations in body weight. This STR not only correlates negatively with body weight but also demonstrates differential expression related to this trait. These results provide valuable insights into the genetic mechanisms regulating growth in shrimp and suggest potential markers for breeding programs aimed at enhancing aquaculture productivity. Additionally, our study introduces a model for STR copy number regulation in non-human species, contributing to the broader understanding of the impact of STRs on complex traits in agricultural animals.

## 1. Introduction

Penaeid shrimp belong to Penaeidae, a family of marine crustaceans in the suborder Dendrobranchiata, which includes many economically important species, such as Pacific white shrimp *Litopenaeus vannamei*, kuruma prawn *Marsupenaeus japonicus*, and the Chinese white shrimp *Fenneropenaeus chinensis* [1,2,3]. The penaeid shrimp has a worldwide distribution in the tropical and subtropical seas, where they constitute an important exploitable resource in estuarine and coastal habitats. Members of the penaeid shrimp have been reported to possess a high percentage of repetitive sequences, with the *Litopenaeus vannamei*, for instance, having repetitive sequences that account for approximately 78% of its genome. Among these, short tandem repeats (STRs) are prominently represented, constituting 10.52–23.93% of the genome [4,5,6]. This high prevalence of STRs positions the penaeid shrimp as an ideal model for studying the genetic and functional roles of STRs. To date, a thorough investigation of the distribution of STRs across multiple species has been largely unexplored.

Short tandem repeats (STRs), also known as microsatellites, are DNA sequences composed of short tandemly repeated units of one to six base pairs [7], which have historically been categorized as nonfunctional DNA [5]. Recent scientific studies have illuminated the direct correlation between STRs and various traits across multiple species [8,9,10,11]. More than 40 human diseases [12], including Huntington’s disease, have been associated with STR expansions, particularly the variation in CAG repeats within the HTT gene [13]. In plants, STRs have been identified as key factors in disease resistance [14,15]. STRs can exert an influence on gene expression and, by extension, phenotypes through a variety of mechanisms [16]. For example, STRs have the capacity to modulate eukaryotic gene expression by altering the binding affinity of transcription factors [17]. Yet, within the context of penaeid shrimp or other crustacean species, the extent to which STRs influence the biological underpinnings of various traits is a subject that has not been deeply probed.

Analysis of the relationship between STRs and transcriptional expression across 17 distinct human tissues has elucidated how STR expansions can broadly impact complex traits, such as height and intelligence, by regulating the expression of proximal genes [9]. Melissa Gymrek’s team proposed a model wherein an increase in the copy number of a single STR could enhance gene expression, thereby affecting the phenotype [9]. Similar observations have been made in the Pacific white shrimp, where individuals with fewer copies of the STR (CT)n in the 5′ UTR region of the LvIRF gene showed higher resistance to the White Spot Syndrome Virus (WSSV) and Decapod Iridescent Virus 1 (DIV1) [18,19]. Notably, offspring selectively bred based on this STR marker demonstrated significant resistance to both viruses, indicating the stable inheritance of this resistance trait. This provides practical evidence supporting the role of STRs in dissecting complex traits of penaeid shrimp.

In this study, we systematically explored the distribution of STRs across multiple species, aiming to utilize the Pacific white shrimp as a model organism for investigating the biological functions of STRs, with a particular focus on growth traits as a case study. We established a high-throughput STR-calling pipeline, which enabled a systematic exploration and assessment of the application of STR markers across the entire genome. Utilizing a mixed linear model in the genome-wide association study (GWAS), we pinpointed growth-related causal STRs and integrated transcriptomic gene expression data to gain preliminary insights into the regulatory mechanisms of STRs that influence growth traits. This work provides a valuable reference for the mining and application of STRs in the biological interpretation of traits in species with high STR content, such as crustaceans, contributing to a deeper understanding of the genetic architecture underlying complex traits in crustacean biology.

## 2. Materials and Methods

### 2.1. Sample Collection and Sequencing

The experimental samples in this study were sourced from Zhanjiang Guangdong Haimao Co., Ltd. A total of 2014 shrimps from 93 families were cultivated under identical experimental conditions in two tanks, with the water temperature maintained at 30 °C and the water depth set to 60 cm. The initial body weight (IBW) of these shrimps was recorded between 101 and 113 days after birth. The final harvested body weight was recorded between 177 and 189 days post-hatch (see Table S1 for sample details). From the population, 5 to 6 individuals were randomly selected from sixty families, yielding a sample of 326 individuals that were evenly distributed across both genders for genomic resequencing. Muscle tissues from the first abdominal segment of each of the 326 individuals were separately harvested for DNA extraction. Library construction and sequencing were carried out on the BGI T7 platform, following the official guidelines. Upon acquisition of the sequencing data, data quality control was conducted using TrimGalore [20] with default parameters to obtain clean data.

### 2.2. STR Identification and Analysis Across Multiple Species

We collected reference genome sequences and protein-coding amino acid sequences of 22 species from NCBI Genbank, including *Litopenaeus vannamei*, *Marsupenaeus japonicu*, *Fenneropenaeus chinensis*, *Procambarus clarkii*, *Cherax quadricarinatus*, *Eriocheir sinensis*, *Portunus trituberculatus*, *Danio rerio, Larimichthys crocea*, *Salmo salar*, *Meretrix meretrix*, *Chlamys farreri*, *Crassostrea gigas*, *Homo sapiens*, *Mus musculus*, *Sus scrofa*, *Poephila guttata*, *Anas platyrhynchos*, *Gallus gallus*, *Oryza sativa*, *Triticum aestivum*, and *Zea mays* (see Table S2 for their reference genome information). Preliminary STR identification was performed on their reference genome sequences using the misa.pl method with default parameters, forming STR coordinate files. The STR identification standards adopted were as follows: the minimum length of the STR region should be at least 10 base pairs, and the minimum repetition number should be 3 times. Specifically, for dinucleotides to hexanucleotides, their minimum repetition times should be 5, 4, 3, 3, and 3 times, respectively. The number and density of STRs were calculated using PySSRstat/statistics_misa.py. The protein-coding amino acid sequences were input into Orthofinder v2.4.1 software for gene family analysis, inferring maximum likelihood species trees from multiple sequence alignments (MSAs). By default, MAFFT is used with default parameters for the alignment and FastTree for the tree inference. The tree were visualized using iTOL v7 software.

### 2.3. STR Calling

We compared and evaluated other reference genomes [21] of *Litopenaeus vannamei* and selected the version [6] that is currently well-annotated and has recently been widely applied in STR identification and functional studies [5]. Using this genome, a comprehensive catalog of 10,453,949 STRs has been established, delineating the precise coordinates of the targeted STR loci. We established a high-throughput STR-calling pipeline based on second-generation short-read sequencing data. Briefly, the second-generation sequencing data were filtered and then aligned to the reference genome using bwa v0.7.17 software to obtain BAM files. Next, using HipSTR v1.0.0 with the parameters specified, --bams represents the input BAM files, --fasta specifies the reference genome file, --regions corresponds to the STR coordinate file, and --str-vcf generates the output VCF file. This approach allowed us to perform targeted STR analysis, resulting in a genotyping VCF file that records the relative copy number of STRs for each sample. The relative copy number is the difference in the number of copies of an STR compared to the reference genome at the same locus. STR filtering was conducted under three conditions: (i) the posterior is < 90%, (ii) more than 15% of reads have a flank indel, or (iii) more than 15% of reads have a stutter artifact. STRs that passed these filtering criteria were defined as high-quality STRs. Finally, we assessed STR variations for the Polymorphism Information Content (PIC) value via a custom script, evaluating the proportions found in low, medium, and high polymorphic sites. The STR sites, originating from 293 individuals with complete phenotypes and demonstrating a presence rate of more than 0.2, were selected for the downstream GWAS analysis.

### 2.4. SNP Calling

The BAM files derived from the preceding genome alignment procedures were subjected to SNP identification using the GATK4 toolkit. In brief, BAM files were used by the GATK HaplotypeCaller engine with the option “-ERC GVCF” to output variants as the genomic variant call format (gVCF). The gVCFs of each sample were merged to conduct a multisample joint genotyping procedure using the GATK GenotypeGVCFs engine. Variant filtering was conducted using the GATK VariantFiltration engine with GATK best practice hard filter guidelines [22]. The additional filtering steps were performed with Plink [23] to retain SNPs with a genotyping rate >80% and to exclude genotypes with a minor allele frequency (MAF) of less than 0.01.

### 2.5. GWAS Analysis

The association analysis was performed using genome-wide STR markers with ASReml 4.2 [24]. The mixed linear model used was the following model:

(1) $$y=1_{n}\mu +Xb+Su+Za+e$$

where y is a vector of observations for the harvested body weight; $1_{n}$ is a vector of ones and μ is the population mean; X is an incidence matrix; b is a vector of factors that include the fixed effect of sex, the covariate of the top five principal components, and the initial body weight nested in the tank; S is a covariate matrix of the STR genotype coded as the sum of the relative copy numbers of two STR alleles; u is a vector of the STR effect; Z is an incidence matrix that links the observations to the random additive genetic effects; a is the vector of random additive genetic effects distributed as $a\sim N(0,G\sigma_{a}^{2})$, where $\sigma_{a}^{2}$ is the additive genetic variance and G is the genomic relationship matrix constructed according to the VanRaden method using STR sites with a missing rate of less than 0.1; and e is a vector of random residual effects distributed as $e\sim N(0,I\sigma_{e}^{2})$, where $\sigma_{e}^{2}$ is the residual variance and I is the identity matrix. After GWAS, using a relatively lenient *p*-value threshold, STRs with *p*-values less than 5 × 10^−4^ were defined as suggestive of significantly associated markers for downstream correlation analyses. We used the previously reported method to calculate the Phenotypic Variance Explained (PVE) for the significant STRs. The Hardy–Weinberg equilibrium (HWE) test for significantly associated STRs was performed using Plink [23]. Significant STRs were then annotated using snpeff to obtain genes around these STRs.

### 2.6. Correlation Analysis

We performed a Pearson correlation analysis on the significant STRs, filtering for correlations with an adjusted harvest body weight above 0.5 or below −0.5. The adjusted harvested body weight was calculated using $\overset{*}{y_{j}}=\hat{a_{j}}+\hat{e_{j}}$, where $\overset{*}{y_{j}}$ is the adjusted phenotype for the harvested body weight of the *j*th individual; $\hat{a_{j}}$ is the estimated breeding value of the *j*th individual; and $\hat{e_{j}}$ is the residual value of the *j*th individual. $\hat{a_{j}}$ and $\hat{e_{j}}$ were obtained from the following model:

(2) $$y_{ij}=\mu +Sex_{i}+\beta bw_{j}(Sex_{i})+a_{j}+e_{ij}$$

where $y_{ij}$ is the harvested body weight of the *j*th individual; μ is the overall mean; $Sex_{i}$ is the fixed effect of the *i*th sex (male or female); $bw_{j}(Sex_{i})$ is the linear covariate of IBW of the *j*th individual nested within $Sex_{i}$; β is regression coefficient for $bw_{j}$; $a_{j}$ is the random additive genetic effect of *j*th individual, $a\sim N(0,A\sigma_{a}^{2})$, where A is the numerator relationship matrix and $\sigma_{a}^{2}$ is the additive genetic variance; $e_{ij}$ is the random residual effect of the *j*th individual, $e\sim N(0,I\sigma_{e}^{2})$, where I is the identity matrix and $\sigma_{e}^{2}$ is the residual variance.

### 2.7. Transcriptome Analysis

An independent population of Pacific white shrimp was utilized for transcriptome analysis. The population was cultivated in BLUP Aquaculture Technology Co., Ltd (Weifang, China). For the experiment, 1440 shrimps from 40 shrimp families were co-cultivated in the same environment. Each shrimp was marked with VIE tags, and, subsequently, these groups were randomly allocated to 40 net cages, each measuring 60 cm in diameter and 80 cm in height, with an effective water volume of 0.17 m^3^. After 55 days of growth, the shrimp were harvested over the next 8 days. Randomly selecting 9 shrimps from each family, a total of 360 individuals were chosen. From these, 80 shrimps, each from the high body weight (HBW) and low body weight groups (LBW), were selected for subsequent transcriptome sequencing. Muscle tissues from the first abdominal segment of each individual were separately collected for RNA extraction. Total RNA was extracted using the TRlzol Reagent (Life Technologies, Carlsbad, CA, USA) manual. Sequencing analysis was performed by Biomarker Technologies Co., Ltd. (Qingdao, China). Sequencing libraries were generated using the Hieff Dual mRNA Kit (Yeasen Biotechnology Co., Ltd., Shanghai, China), and each library was sequenced on the Illumina NovaSeq platform.

Clean data were obtained by removing sequences containing adapters, sequences containing polyN, and low-quality sequences from the raw data using internal Perl scripts. After quality control, the clean data were aligned to the reference genome sequence using HISAT2 [25] with the parameters “--dta -p 6 --max-intronlen 5,000,000” to ensure accurate read mapping and transcript identification. The aligned reads were then assembled into transcripts using StringTie [26], enabling accurate reconstruction and quantification of gene and transcript expression. Gene and transcript expression levels were normalized using FPKM (fragments per kilobase of transcript per million fragments mapped) [27] to account for variations in sequencing depth and transcript length. Subsequently, Prism was used for differential gene expression analysis to screen for significantly differentially expressed genes, employing a *t*-test model for statistical evaluation.

## 3. Results

### 3.1. Penaeid Shrimp Exhibit a Rich Content of STRs

**Figure 1:**
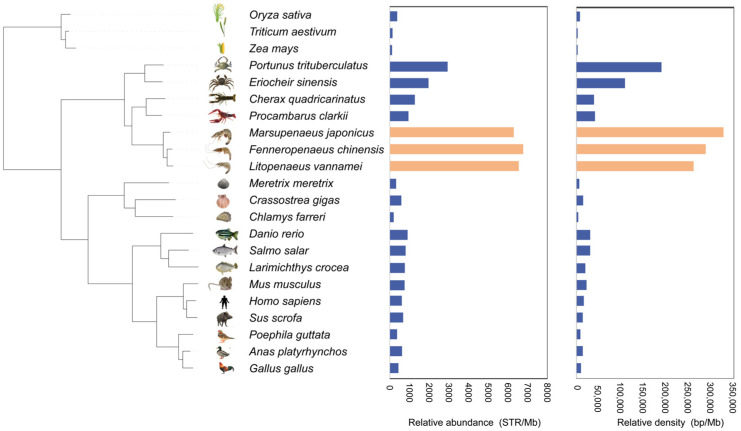
The evolutionary relationship of 22 species is illustrated, with a detailed distribution of the relative density and the relative content of STRs across their genomes. The orange bars represent species from the Penaeidae family, the blue bars represent species from other family.

STR identification and comparison were conducted across the genomes of 22 diverse species, encompassing a wide range of taxa including crustaceans, fish, shellfish, mammals, birds, and plants. Among the Penaeidae family, three penaeid shrimp, *Fenneropenaeus chinensis*, *Marsupenaeus japonicus*, and *Litopenaeus vannamei*, show an exceptionally high frequency of STRs. Their genomes contain 9,583,760, 11,531,166, and 10,453,949 STRs, respectively, with a relative abundance of 6537 to 6763.1 per megabase (Mb). Their STR density is also high, ranging from 260,102 to 326,550 base pairs per megabase (Mb), which corresponds to 26% to 32% of the total sequence in each species, as shown in Figure 1 and detailed in Table S3. 

This density is markedly higher than that observed in other crustaceans, such as *Procambarus clarkii*, *Cherax quadricarinatus*, *Eriocheir sinensis*, and *Portunus trituberculatus*, which have STR genome proportions ranging from 4.1% to 18%. Similarly, shellfish species like *Meretrix meretrix*, *Chlamys farreri*, and *Crassostrea gigas* exhibit lower STR proportions of 0.36–1.44%. The lowest STR genome proportions were observed in the plant species *Zea mays* (0.21%), *Triticum aestivum* (0.24%), and *Oryza sativa* (0.73%), as detailed in Table S3. Mammals, such as *Homo sapiens*, *Sus scrofa*, and *Mus musculus*, exhibit moderate genomic STR content, ranging from 1.3% to 2.1%.

### 3.2. Dinucleotide Repeats Dominate the STRs in Penaeid Shrimp

**Figure 2:**
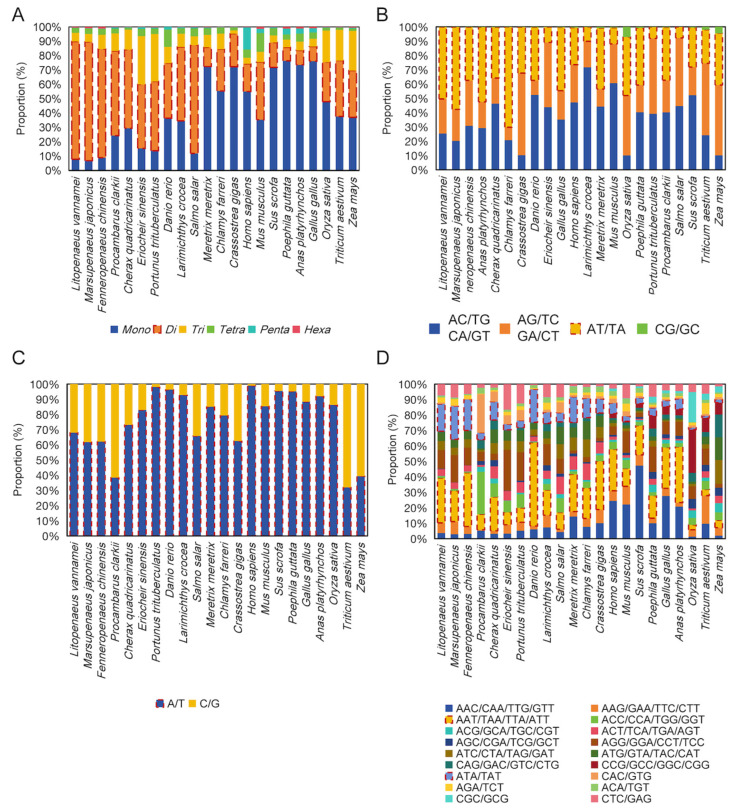
(**A**) Proportions of STRs with 1–6 base units in 22 species. (**B**–**D**) The proportion of mononucleotide, dinucleotide, and trinucleotide STRs present in 22 species.

We conducted an in-depth analysis of the distribution of STRs with one to six nucleotides among 22 species. Notably, penaeid shrimp has a significantly higher proportion of dinucleotide repeats, constituting over 70% of the total STR count, except for *Salmo salar* at 75.4% (Figure 2A, Table S4). Among the other species, birds exhibit the lowest proportion of dinucleotide repeats, averaging around 10% (Figure 2A, Table S4). However, birds, shellfish, and mammals predominantly feature mononucleotide repeat sequences. Interestingly, the two crab species, which are also crustaceans, have the highest content of trinucleotide repeats, both surpassing 28%. The crayfish species show a similar pattern to penaeid shrimp, particularly with a higher prevalence (59.5–70.38%) of dinucleotide repeats (Figure 2A, Table S4).

Further analysis of the composition of dinucleotide repeats revealed that the dinucleotide repeats in *Litopenaeus vannamei*, *Marsupenaeus japonicus*, and *Fenneropenaeus chinensis* are dominated by AT/TA repeats, accounting for 50.3% (8,646,248), 57.6% (11,017,520), and 37.3% (5,399,092), respectively. This proportion is only 7.9% (331,554) and 10.8% (225,696) in *Eriocheir sinensis* and *Portunus trituberculatus*, respectively (Figure 2B, Table S5). However, these two crab species have a high proportion of AG/GA repeats at 22.6% (695,386) and 26.3% (748,002), respectively. This figure is significantly higher than the 8.9–15.8% found in penaeid shrimp and two crayfish species (Figure 2B, Table S5a). Similarly, the trinucleotide repeats (AAT/TAA/TTA/ATT/ATA/TAT) in the two penaeid shrimp species, *Litopenaeus vannamei* (50.34%) and *Marsupenaeus japonicus* (57.64%), make up about half of the total trinucleotide repeats (Figure 2C, Table S5b). While single A/T base repeats dominate the mononucleotide repeats in penaeid shrimp, constituting 61.8% to 68%, these repeats are even more prevalent in mammals, shellfish, and birds, exceeding 85% (Figure 2D, Table S5c). The abundance of STR repeats composed of the A or T base suggests that they may potentially have a regulatory role in the penaeid shrimp genome.

### 3.3. STRs Demonstrate Significant Potential as Associative Markers

**Figure 3:**
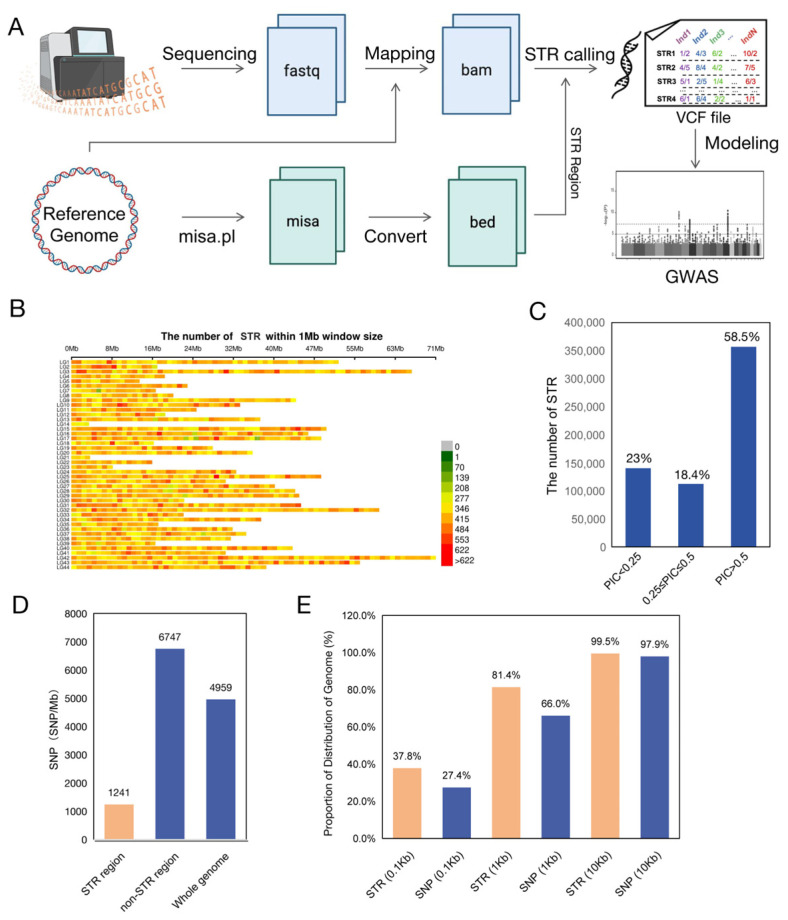
(**A**) Workflow for high-throughput STR identification analysis based on second-generation sequencing data. (**B**) Heatmap of STR marker distribution across the whole genome. (**C**) Proportion of distribution of STR sequence lengths. (**D**) Density of SNPs in the genome STR region, non-STR region, and whole genome region. The STR regions are represented in yellow, while the other two groups are represented in blue. (**E**) Evenness of STR and SNP distribution across the whole genome, with statistics taken in 0.1 Kb, 1 Kb, and 10 Kb windows. Red and blue represent significantly expressed genes and non-significantly expressed genes, respectively.

To further explore the roles and potential of STRs in the genome of penaeid shrimp, we used the 10,453,949 STRs from the *Litopenaeus vannamei* reference genome to create a genomic coordinate reference. With this reference, we built a high-throughput detection pipeline tailored for identifying STRs from second-generation sequencing data (Figure 3A). Resequencing data from 326 *Litopenaeus vannamei* individuals revealed 672,507 evenly distributed STR markers across the genome (Figure 3B). Information on these population STRs, including the distribution of allele count differences from the reference allele for STRs, total STR length distribution by repeat period length, and allele count distribution for STR loci, was also calculated and presented in Figure S1. These STRs are likely common STRs, while other STRs not found in the population may be considered rare STRs. Notably, 58.5% of these markers, totaling 356,689, have a high polymorphic information content (PIC) value above 0.5 (Figure 3C). The markers’ high polymorphism, even distribution, and abundance suggest that STRs are promising molecular markers for selective breeding in penaeid shrimp.

We further compared the distribution and density of STRs and SNPs genome-wide and identified 8,249,898 high-quality SNPs within the same cohort. We found that the SNP density in regions containing STRs was about one-fifth of that in STR-free regions, with a ratio of 1241 SNPs to 6747 SNPs (Figure 3D). This suggests that the 26% of the *Litopenaeus vannamei* genome rich in STRs has a lower SNP content. Relying only on SNPs as molecular markers might therefore overlook genetic effects in these STR-rich areas.

Additionally, we evaluated the distribution uniformity of STR and SNP markers across the genome. Comparing their presence in 0.1 Kb, 1 Kb, and 10 Kb non-overlapping windows, STRs demonstrated higher occupancy rates of 37.8%, 81.4%, and 99.5%, respectively, compared to SNPs at 27.4%, 66.0%, and 97.9% (Figure 3E). This indicates that STRs are more evenly distributed than SNPs at all assessed scales, likely due to their greater abundance in the *Litopenaeus vannamei* genome.

### 3.4. Association and Correlation Analysis of STRs and Growth Traits

**Figure 4:**
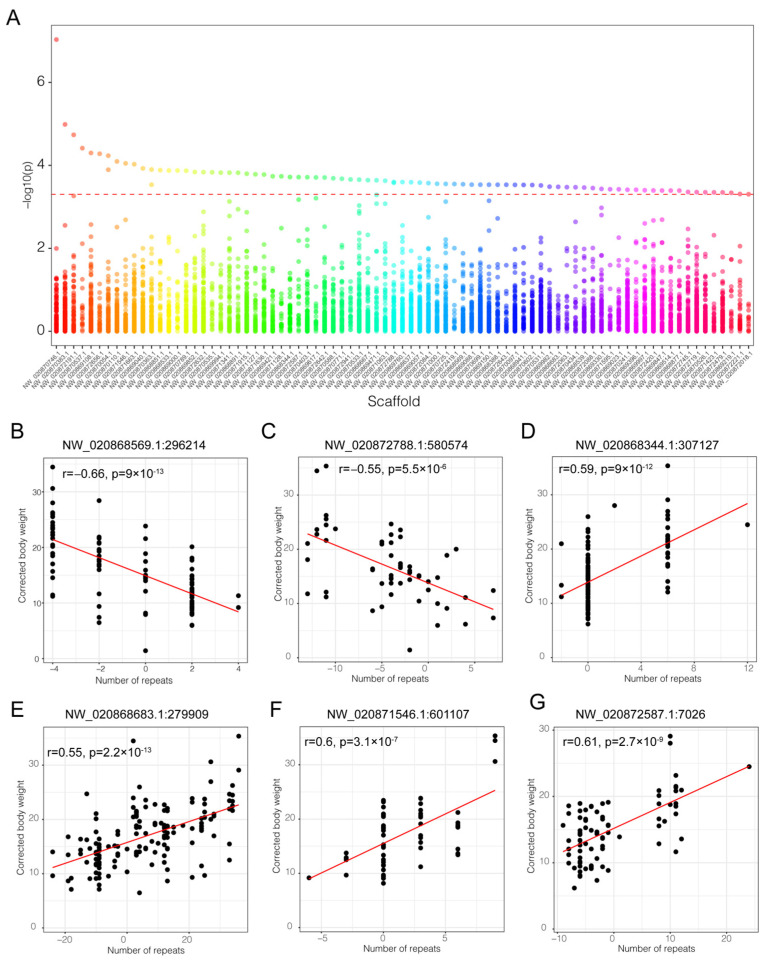
(**A**) Manhattan plot of the 84 significant STR loci. (**B**–**G**) Scatter plots showing the correlation analysis between adjusted weight and relative copy number of significantly associated STRs (|r| > 0.55).

To uncover molecular markers related to growth traits in *Litopenaeus vannamei*, we conducted a GWAS focusing on STR copy number variations and their correlation with adjusted harvest weight. Analyzing 173,433 STR loci, we identified 84 with significant associations to the phenotype (*p* < 5 × 10^−4^) (Figure 4A, Table S6). Among these 84 STRs, only three sets of 2 STRs were located on the same genomic scaffold, while the remaining STRs were distributed across different scaffold sequences. Four significantly associated STRs, NW_020871546.1:601107, NW_020870537.1:94961, NW_020869108.1:710311, and NW_020872788.1:580574, have PVE values greater than 0.2. Notably, NW_020871546.1:601107 has the highest PVE value of 0.23 (Table S6), indicating that these STRs have a high explanatory power for the phenotype. Out of the 84 significantly associated STRs, 71 STRs did not show a significant deviation from the Hardy–Weinberg equilibrium (*p* > 0.05) (Table S6). The PIC values of these significant STRs ranged from 0.352 to 0.8454, indicating high polymorphism (Table S6). In these associated STRs, we found that 28 (33.33%) were mononucleotides, 24 (28.57%) were dinucleotides, 26 (30.95%) were trinucleotides, and 6 (7.14%) were tetranucleotides. Strikingly, more than three-fifths of these significantly associated STRs—specifically, 63% (53 out of 84)—contained repeat units based on an A/T base. The single A base repeat was particularly prominent, accounting for 21.4% (18 out of 84) of the associated STRs, with the single T base repeat following at 11.9% (10 out of 84). This high incidence suggests that STRs rich in A/T repeats may play a pivotal role in the regulation of the growth trait in shrimp (Table 1). Functional analysis revealed that these 84 associated STRs were annotated to 95 genes, including 1-acyl-sn-glycerol-3-phosphate acyltransferase alpha-like (LOC113829252), nuclear receptor subfamily 2 group E member 1-like (LOC113806342), syndecan-3-like (LOC113807065), mucin-2-like (LOC113815059), poly [ADP-ribose] polymerase 3-like (LOC113815759), huntingtin-like (LOC113820996), and syndecan-3-like (LOC113807065).

The correlation analysis of the 84 significantly associated STRs with the adjusted harvest body weight phenotype revealed that 11 STRs demonstrated a significant linear correlation (|r| > 0.5, Table 1 and Table S7, Figure 4). A striking 81% (9 out of 11) of these STRs contained repeat units based on an A/T base, with five single A/T unit repeats. The STR at the locus NW_020868569.1:296214 showed the highest negative correlation of −0.66 (*p* = 9 × 10^−13^), with individuals having a maximum of 4 relative copy numbers and an average weight 11.701 g lighter than those with the lowest relative copy number of −4. This locus is an intronic variant of the gene coding for cytoplasmic dynein 1 intermediate chain-like (LOC113822999). Additionally, the locus NW_020871546.1:601107 exhibited the strongest positive correlation with a coefficient of 0.6 (*p* = 3.1 × 10^−7^). Individuals bearing a maximum relative copy number of nine at this locus had an average weight of 33.47 g. This is significantly heavier than individuals who had a minimum relative copy number of −6 at this locus at 25.32 g, who weighed an average of just 9.17 g. This locus is in an intergenic region near the nascent polypeptide-associated complex subunit alpha, the muscle-specific form-like gene (LOC113825946), which is associated with muscle growth [28], suggesting its potential involvement in shrimp growth regulation. Lastly, the STR, NW_020872788.1:58057, which is a splice region variant of the dedicator of cytokinesis protein 7-like (LOC113800912), exhibited a negative correlation of −0.55 (*p* = 5.5 × 10^−6^) with the weight value. Individuals possessing a maximum of 7 relative copies at this locus have an average weight of 9.86 g, which is markedly lighter, by 7.12 g, compared to those with the minimum relative copy number of −13, who weighed an average of 16.98 g. These results suggest that STR copy number variations may possibly play a role in regulating the shrimp’s weight phenotype.

### 3.5. Integration Analysis of Transcriptome Data with Associated STRs

**Figure 5:**
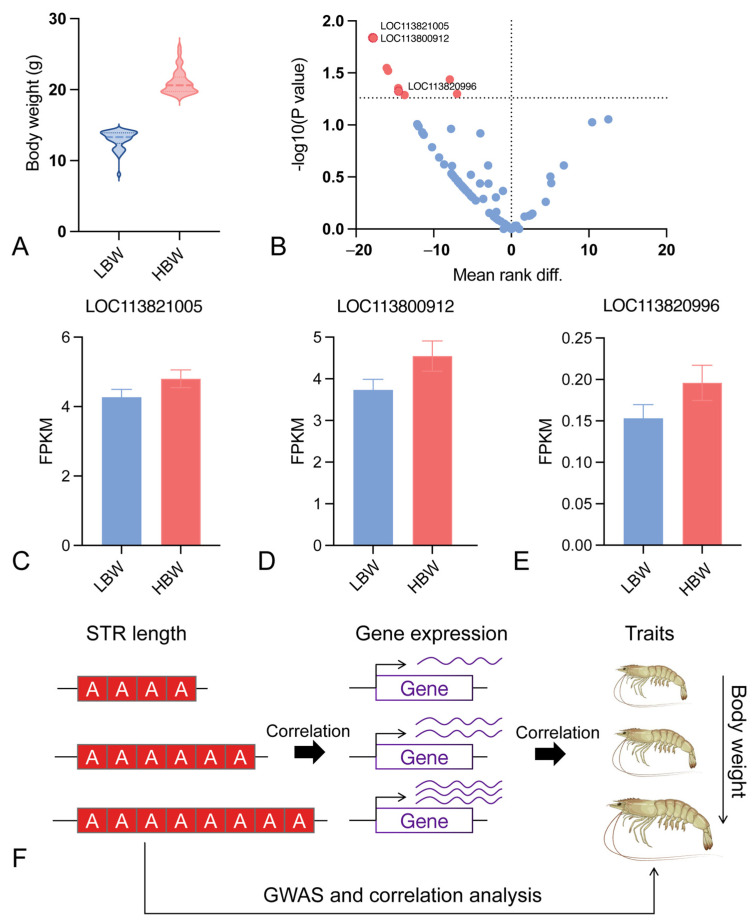
(**A**) Violin plot of the weights of high body weight (HBW) and low body weight (LBW) shrimps used for transcriptome sequencing. Blue and red represent LBW and HBW group, respectively. (**B**) Volcano plot of differential expression of genes annotated by 84 significantly associated STR loci. Red and blue represent significantly expressed genes and non-significantly expressed genes, respectively. (**C**–**E**) Three sets of differentially expressed genes (*p* < 0.05). Blue and red represent LBW and HBW group, respectively. (**F**) The plot shows the regulatory model of the STR copy number regulating gene expression and thus affecting the body weight phenotype in *Litopenaeus vannamei*.

We furthered our investigation with a comparative transcriptome sequencing study utilizing independent populations. Specifically, we selected 80 individuals from both the high body weight (HBW) and low body weight (LBW) groups. On average, the HBW group had body weights that were more than 8 g heavier than those of the LBW group (Figure 5A). Through a thorough differential gene expression analysis, we compared the expression profiles of 95 genes that had been annotated with significant STRs between the HBW and LBW groups. The analysis identified nine genes with significant differential expression (*p* < 0.05, Figure 5B, Table S8): muskelin-like (LOC113821005), huntingtin-like (LOC113820996), myelin regulatory factor-like (LOC113820076), mitochondrial import receptor subunit TOM22 homolog (LOC113815758), cytoplasmic dynein 1 intermediate chain-like (LOC113822999), copine-9-like (LOC113830508), cyclic nucleotide-gated channel cone photoreceptor subunit alpha-like (LOC113809390), dedicator of cytokinesis protein 7-like (LOC113800912), and LOC113807713. Among them, LOC113809390, LOC113800912, and LOC113822999 are annotated with the STRs at the locus of NW_020869421.1:132288 [(TAA)n], NW_020872788.1:580574 [(A)n], and NW_020868569.1:296214 [(AT)n], respectively (Figure 5C–E, Table 1). These loci have exhibited a significant correlation with body weight (Table 1), suggesting a potential role in controlling body weight by modulating the expression of genes associated with weight regulation. Based on these results, we postulate a model in which the copy number variation of STRs may alter gene expression, consequently exerting an influence on the body weight characteristic in penaeid shrimp, as illustrated in Figure 5F. This model suggests that STRs could function as regulatory elements, linearly influencing complex traits through the modulation of genetic expression.

## 4. Discussion

In this study, we observed significant variations in the content of STRs across different species. Notably, within the *Penaeidae* family, species like *Litopenaeus vannamei*, *Marsupenaeus japonicus*, and *Fenneropenaeus chinensis* displayed the highest STR content, ranging from 26% to 32%, which is considerably higher than that observed in other species. Prior studies have revealed that the extreme expansion of STRs in penaeid shrimp genomes may be largely driven by transposable elements (TEs), which serve as carriers for STR propagation [5]. The expansion of STRs in penaeid shrimp genomes is likely tied to two major mass extinction events in the Earth’s history—the Permian–Triassic extinction and the Late Cretaceous extinction. During these periods, STR expansions and species-specific expansions of certain gene families are observed, particularly within the Penaeidae family [5]. These expansions are believed to have contributed to the remarkable genomic plasticity of penaeid shrimp, enabling them to adaptively evolve, rapidly diversify, and occupy new ecological niches following these extinction events [5].

The high prevalence of STRs in Penaeidae is partly attributable to the fact that dinucleotide repeats constitute over 70% of all STRs within their genomes. A previous study in *Drosophila melanogaster* has associated dinucleotide STRs with the extensive activity of cis-regulatory elements across various cell types [29]. A significant number of these dinucleotide STRs are characteristic of broadly active enhancers, suggesting that they may be essential for widespread enhancer activity [29]. Furthermore, dinucleotide STRs are known to be enriched in human regulatory regions, as evidenced by DNase I hypersensitivity and histone modifications, such as H3K4me1 and H3K27ac, according to the ENCODE Project Consortium. The enrichment of dinucleotide STRs suggests a key role in enhancer function across species. In this study, we identified 28 dinucleotide STRs, representing 28.57% of the total, that are significantly associated with body weight. Notably, NW_020868569.1:296214 [(AT)n] has been demonstrated to potentially participate in regulating the expression of the cytoplasmic dynein 1 intermediate chain-like gene, a gene that shows significant differential expression between the high and the low weight groups. These findings suggest that the enhancer activity of these STRs may be a potential mechanism through which they contribute to the regulation of weight traits.

STRs are predicted to contribute a higher number of de novo mutations per generation than any other type of variation [30]. In comparison to mutation rates among different types of STRs, dinucleotide repeats in human STR loci that are without pathological effects exhibit the highest mutation rates, with tetranucleotide repeats showing rates that are 50% lower [31]. Similarly, in *Drosophila melanogaster*, tri- and tetranucleotide repeats are found to mutate at rates that are 6.4 and 8.4 times slower, respectively, than those of dinucleotide repeats. We found that TA/AT dinucleotides, in particular, constitute the majority of dinucleotide repeats in the penaeid shrimp genome. Mutation rates vary significantly among different motif types in *Arabidopsis thaliana*, with AT repeats having the highest mutation rate, followed by CT repeats, and CA repeats exhibiting the lowest rate. They have been identified as a significant destabilizing element. Studies on endogenous transcripts have shown that high TA–dinucleotide ratios in untranslated regions (UTRs) can lead to increased RNA degradation [32]. High levels of dinucleotide repeats, particularly AT repeats, may contribute to the high mutation rate and genetic diversity observed in shrimp genomes. This could be a key factor in the high adaptability of shrimp species to the complex marine environment.

STRs have traditionally been markers of focus on a limited scale, with exploration primarily centered around a few loci [7,33,34]. However, they have seldom been systematically mined and applied across the entire genome. The shrimp genome’s wealth of STRs has opened a new window for their utilization and application. In the study, we identified a total of 672,507 STRs, which were evenly distributed across the genome. Interestingly, we observed a lower incidence of SNPs within these STR regions, potentially due to a decreased accuracy in SNP identification in the repeat region [35]. These observations highlight the potential of STRs in the shrimp genome as powerful associative markers for GWAS. Unlike biallelic SNPs, STRs possess a distinct ability to influence phenotypic variation through a spectrum of multiple alleles. Specifically, 84 STRs were found to be significantly associated with body weight. A subset of these STRs showed a direct linear correlation between various repeat numbers and the phenotype, a relationship that is not readily explained by the presence of nearby biallelic variants. Moreover, genes potentially regulated by these STRs demonstrated significantly differential expression between high and low body weight groups, such as cyclic nucleotide-gated channel cone photoreceptor subunit alpha-like gene, dedicator of cytokinesis protein 7-like gene, and cytoplasmic dynein 1 intermediate chain-like gene.

Building upon the seminal research of Stephanie et al., which illuminated the ways in which STR expansions can modulate the gene expression of proximal genes, thereby influencing complex human traits, such as height and intelligence, we have broadened this line of investigation to include invertebrates, specifically the penaeid shrimp, whose genome is rich in STR sequences. The model indicates that in penaeid shrimp, an increase in the copy number of several STRs can result in alterations of gene expression, which may subsequently affect body weight phenotypes. In line with this model, our study has uncovered 11 significant correlations between variations in the STR copy number and body weight. Notably, 81% of these STRs contained repeat units primarily composed of an A/T base. The AT-rich regions’ structural properties may promote local DNA unwinding and gene expression due to the weaker hydrogen bonding of A/T base pairs, suggesting implications for transcriptional activity in these zones [36,37]. We identified three weight-associated differentially expressed genes, including the cyclic nucleotide-gated channel cone photoreceptor subunit alpha-like gene, the dedicator of cytokinesis protein 7-like gene, and the cytoplasmic dynein 1 intermediate chain-like gene, which appear to be regulated by AT-base-related STRs at NW_020869421.1:132288 (TAA)n, NW_020872788.1:580574 (A)n, and NW_020868569.1:296214 (AT)n, respectively. These findings suggest that A/T-base-related STRs might influence body weight in shrimp by modulating the expression of body-weight-associated genes. In particular, the STR at the locus of NW_020872788.1:58057 is located within the splice region of the dedicator of cytokinesis protein 7-like gene, which demonstrated significant expression differences between high body weight and low body weight groups. A previous study has implicated this gene in pathways related to lipid and lipoprotein metabolism, suggesting a potential contribution of this gene to growth traits in shrimp. Moreover, the STR at the locus of NW_020870699.1:1693451 is sited in the intergenic region between the huntingtin-like gene and the muskelin-like gene, both of which exhibited notable differences in expression across body weight groups. The huntingtin gene, in particular, has been associated with body weight in multispecies in previous studies [38,39,40], possibly via its regulatory effects on the insulin-like growth factor. Overall, these findings provide preliminary insights into the regulatory mechanisms potentially underlying growth traits in shrimp, thereby highlighting the applicability of the STR regulation model in the context of penaeid shrimp.

In this study, we utilized a previously published and widely used reference genome of *Litopenaeus vannamei* [6], which has recently been extensively applied in STR identification and functional studies [5,18,19]. The STRs identified using this genome were validated for genotypic polymorphism in resequencing data (n = 326), confirming their reliability. Recently, the genome of *Litopenaeus vannamei* has been updated, and the new version shows improvements in genome integrity compared to the previous assembly [21]. However, the updated genome has not yet been provided with official NCBI gene annotations, which are critical for transcriptome analysis and the identification of candidate genes and regulatory mechanisms related to growth traits. Additionally, the new genome has a slightly lower BUSCO completeness score and exhibits more fragmentation compared to the previous version, which may affect candidate gene identification and STR detection [6,21]. As a result, the new genome version is currently less suitable for this study. The updated genome represents a valuable contribution to shrimp genomics and will undoubtedly serve as an important resource for future research. The release of more complete gene annotations in the future will enhance STR research and advance the understanding of their biological roles in *Litopenaeus vannamei*.

## 5. Conclusions

In conclusion, this study underscores the substantial presence and significant impact of STRs in penaeid shrimp genomes, which are notably higher than in mammals and plants. With 26–32% of their genomes consisting of STRs, these sequences play a pivotal role in regulating growth traits. Our analysis of 326 Pacific white shrimp identified 672,507 high-quality STRs. A genome-wide association study pinpointed 84 STRs linked to body weight, primarily composed of A/T bases. This includes the [(A)n] repeat at NW_020872788.1:580574, which is significantly associated with body weight and impacts the expression of the cytokinesis protein 7-like gene. These findings provide crucial insights into STR functions and their potential for improving breeding strategies in shrimp and other agricultural animals. The combination of a high STR content and the presence of functional STRs positions Pacific white shrimp as a promising model organism for studying the biological roles of STRs.

## Figures and Tables

**Table 1:** STRs significantly associated with and correlated to the harvested body weight in *Litopenaeus vannamei*.

Scaffold	Pos	Unit	*p* Value	r Value	Gene
NW_020868344.1	307127	(CA)n	1.91 × 10^−4^	0.59	LOC113803219
NW_020868539.1	104464	(A)n	3.43 × 10^−4^	0.52	LOC113820924, LOC113821003
NW_020868569.1	296214	(AT)n	2.85 × 10^−4^	−0.66	LOC113822999
NW_020868683.1	279909	(TAA)n	3.30 × 10^−4^	0.55	LOC113802910
NW_020869000.1	338751	(A)n	1.33 × 10^−4^	0.51	LOC113805488, LOC113805487
NW_020869150.1	290225	(T)n	2.93 × 10^−4^	0.53	LOC113807065
NW_020869421.1	132288	(TAA)n	1.84 × 10^−4^	−0.51	LOC113809390
NW_020870537.1	94961	(AG)n	3.85 × 10^−5^	−0.53	
NW_020871546.1	601107	(TAA)n	8.95 × 10^−5^	0.60	LOC113825946, LOC113825948
NW_020872587.1	7026	(A)n	1.92 × 10^−4^	0.61	
NW_020872788.1	580574	(A)n	2.52 × 10^−4^	−0.55	LOC113800912

## Data Availability

Sequencing data generated for this project have been deposited in the NCBI Sequence Read Archive under the BioProject ID PRJNA1112592.

## Supplementary Materials

The following supporting information can be downloaded at https://www.mdpi.com/article/10.3390/ani15020262/s1, Figure S1: (A) Distribution of allele sizes relative to the reference genome for STRs. The x-axis represents the difference in allele sizes compared to the reference genome, with positive values indicating longer alleles and negative values indicating shorter alleles relative to the reference. The y-axis shows the number of alleles on a logarithmic scale. (B) Total STR length distribution by repeat period length. The x-axis indicates the STR period length (1-6), and the y-axis represents the total length of STRs in base pairs for each period. (C) Allele count distribution for STR loci. The x-axis denotes the allele count for each STR, and the y-axis shows the frequency of occurrences in the population; Table S1: The Information of samples used in this study; Table S2: The Information of 22 species genomes used in this study.; Table S3: The relative density and abundance of STRs in 22 species; Table S4a: The number distribution of six types of STRs in 22 species; Table S4b: The lenght distribution of six types of STRs in 22 species; Table S5: a: The number and proportion of mononucleotides STR in 22 species; b: The number and proportion of dinucleotides STR in 22 species; c: The number and proportion of trinucleotides STR in 22 species; Table S6: The significant STRs related harvest body weight; Table S7: The significant STRs demonstrated a significant linear correlation with harvest body weight; Table S8: a: The expression of genes located in genes annotated by STRs significantly associated with harvest body weight; b: The differential expression analysis of genes located in genes annotated by STRs significantly associated with harvest body weight.
