# Supplementary figures and images for: Copy Number Variations in Short Tandem Repeats Modulate Growth Traits in Penaeid Shrimp Through Neighboring Gene Regulation
Source: Animals (Basel). 2025 Jan 18;15(2):262. doi: 10.3390/ani15020262 (PMC11758629; doi:10.3390/ani15020262)

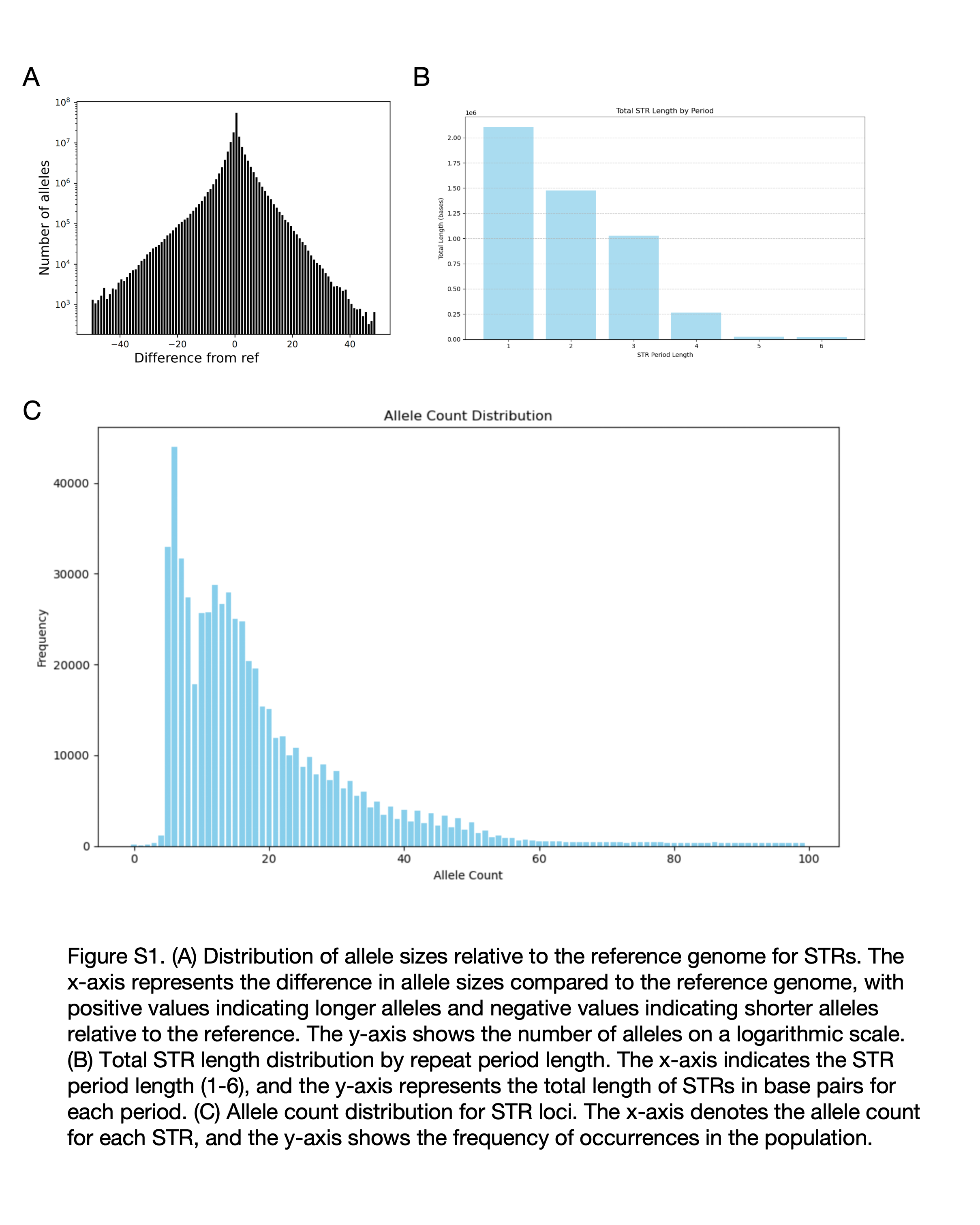

Supplement: Supplementary file 1 [file animals-15-00262-s001.zip › Figure S1.tif]
